# Publisher’s Note: We Changed Page Numbers to Article Numbers for Articles Published in *Clinics and Practice* Volumes 1–10

**DOI:** 10.3390/clinpract12020019

**Published:** 2022-02-23

**Authors:** Clinics and Practice Editorial Office

**Affiliations:** MDPI AG, St. Alban-Anlage 66, 4052 Basel, Switzerland; clinpract@mdpi.com

*Clinics and Practice* [1] was published by PAGEPress from Volume 1 (2011) to Volume 10 (2020). From Volume 11, Issue 1 (2021), *Clinics and Practice* is published by MDPI.

Previous articles in Volumes 1–10, published by PAGEPress in Open Access under a CC-BY (or CC-BY-NC-ND) licence, are now hosted by MDPI on mdpi.com as a courtesy and upon agreement with PAGEPress.

To standardize the metadata format of all previous articles, MDPI republished 513 articles in Volumes 1–10 with article numbers replacing page numbers (Table 1).

## Figures and Tables

**Table 1:** MDPI changed page numbers to articles numbers for 513 articles in Volumes 1–10.

DOI	Previous Page Number	Current Article Number
10.4081/cp.2011.e1	1–2	e1
10.4081/cp.2011.e2	3	e2
10.4081/cp.2011.e3	4–5	e3
10.4081/cp.2011.e4	6	e4
10.4081/cp.2011.e5	7–8	e5
10.4081/cp.2011.e6	9–10	e6
10.4081/cp.2011.e7	11–13	e7
10.4081/cp.2011.e8	14	e8
10.4081/cp.2011.e9	15–16	e9
10.4081/cp.2011.e10	17	e10
10.4081/cp.2011.e11	18–19	e11
10.4081/cp.2011.e12	20–21	e12
10.4081/cp.2011.e13	22–23	e13
10.4081/cp.2011.e14	24	e14
10.4081/cp.2011.e15	25	e15
10.4081/cp.2011.e16	26–27	e16
10.4081/cp.2011.e17	28–29	e17
10.4081/cp.2011.e18	30	e18
10.4081/cp.2011.e19	31–32	e19
10.4081/cp.2011.e20	33–34	e20
10.4081/cp.2011.e21	35–36	e21
10.4081/cp.2011.e22	37–38	e22
10.4081/cp.2011.e23	39–40	e23
10.4081/cp.2011.e24	41–42	e24
10.4081/cp.2011.e25	43–45	e25
10.4081/cp.2011.e26	46–47	e26
10.4081/cp.2011.e27	48–49	e27
10.4081/cp.2011.e28	50–51	e28
10.4081/cp.2011.e29	52–53	e29
10.4081/cp.2011.e30	54–55	e30
10.4081/cp.2011.e31	56–58	e31
10.4081/cp.2011.e32	59–60	e32
10.4081/cp.2011.e33	61–62	e33
10.4081/cp.2011.e34	63–64	e34
10.4081/cp.2011.e35	65–66	e35
10.4081/cp.2011.e36	67–69	e36
10.4081/cp.2011.e38	72–73	e38
10.4081/cp.2011.e39	74–75	e39
10.4081/cp.2011.e40	76	e40
10.4081/cp.2011.e41	77–78	e41
10.4081/cp.2011.e42	79–81	e42
10.4081/cp.2011.e43	82–83	e43
10.4081/cp.2011.e44	84–87	e44
10.4081/cp.2011.e45	88–90	e45
10.4081/cp.2011.e46	91–93	e46
10.4081/cp.2011.e47	94–95	e47
10.4081/cp.2011.e48	96–97	e48
10.4081/cp.2011.e49	98–99	e49
10.4081/cp.2011.e50	100–101	e50
10.4081/cp.2011.e51	102–104	e51
10.4081/cp.2011.e52	105–106	e52
10.4081/cp.2011.e53	107–109	e53
10.4081/cp.2011.e54	110	e54
10.4081/cp.2011.e55	111–113	e55
10.4081/cp.2011.e56	114–115	e56
10.4081/cp.2011.e57	116–118	e57
10.4081/cp.2011.e58	119–120	e58
10.4081/cp.2011.e59	121–122	e59
10.4081/cp.2011.e60	123–125	e60
10.4081/cp.2011.e61	126–127	e61
10.4081/cp.2011.e62	128–129	e62
10.4081/cp.2011.e63	130–131	e63
10.4081/cp.2011.e64	132–133	e64
10.4081/cp.2011.e65	134–135	e65
10.4081/cp.2011.e66	136–138	e66
10.4081/cp.2011.e67	139–141	e67
10.4081/cp.2011.e68	142–143	e68
10.4081/cp.2011.e69	144	e69
10.4081/cp.2011.e70	145–146	e70
10.4081/cp.2011.e71	147–148	e71
10.4081/cp.2011.e72	149–150	e72
10.4081/cp.2011.e73	151–152	e73
10.4081/cp.2011.e74	153–154	e74
10.4081/cp.2011.e75	155–156	e75
10.4081/cp.2011.e76	157–158	e76
10.4081/cp.2011.e77	159–161	e77
10.4081/cp.2011.e78	162–163	e78
10.4081/cp.2011.e79	164–166	e79
10.4081/cp.2011.e81	169–171	e81
10.4081/cp.2011.e82	172–173	e82
10.4081/cp.2011.e83	174–178	e83
10.4081/cp.2011.e84	179	e84
10.4081/cp.2011.e85	180–181	e85
10.4081/cp.2011.e86	182–184	e86
10.4081/cp.2011.e87	185–186	e87
10.4081/cp.2011.e88	187–188	e88
10.4081/cp.2011.e89	189–192	e89
10.4081/cp.2011.e90	193–194	e90
10.4081/cp.2011.e91	195–197	e91
10.4081/cp.2011.e92	198–199	e92
10.4081/cp.2011.e93	200–202	e93
10.4081/cp.2011.e94	203–204	e94
10.4081/cp.2011.e95	205–208	e95
10.4081/cp.2011.e96	209–210	e96
10.4081/cp.2011.e97	211–212	e97
10.4081/cp.2011.e98	213–214	e98
10.4081/cp.2011.e99	215–217	e99
10.4081/cp.2011.e100	218–221	e100
10.4081/cp.2011.e101	222–223	e101
10.4081/cp.2011.e102	224–225	e102
10.4081/cp.2011.e103	226–227	e103
10.4081/cp.2011.e104	228–229	e104
10.4081/cp.2011.e105	230–231	e105
10.4081/cp.2011.e106	232–233	e106
10.4081/cp.2011.e107	234–236	e107
10.4081/cp.2011.e108	237–238	e108
10.4081/cp.2011.e109	239	e109
10.4081/cp.2011.e110	240	e110
10.4081/cp.2011.e111	241–242	e111
10.4081/cp.2011.e112	243–244	e112
10.4081/cp.2011.e113	245–246	e113
10.4081/cp.2011.e114	247–248	e114
10.4081/cp.2011.e115	249–250	e115
10.4081/cp.2011.e116	251–252	e116
10.4081/cp.2011.e117	253–255	e117
10.4081/cp.2011.e118	256–258	e118
10.4081/cp.2011.e119	259–260	e119
10.4081/cp.2011.e120	261–262	e120
10.4081/cp.2011.e121	263–264	e121
10.4081/cp.2011.e122	265–266	e122
10.4081/cp.2011.e123	267–268	e123
10.4081/cp.2011.e124	269–273	e124
10.4081/cp.2011.e125	274–276	e125
10.4081/cp.2011.e126	277–278	e126
10.4081/cp.2011.e127	279–281	e127
10.4081/cp.2011.e128	282–283	e128
10.4081/cp.2011.e129	284–286	e129
10.4081/cp.2011.e130	287–289	e130
10.4081/cp.2011.e131	290–291	e131
10.4081/cp.2011.e132	292–295	e132
10.4081/cp.2011.e133	296–298	e133
10.4081/cp.2011.e134	299–301	e134
10.4081/cp.2011.e135	302–303	e135
10.4081/cp.2011.e136	304–305	e136
10.4081/cp.2011.e137	306–307	e137
10.4081/cp.2011.e138	308–312	e138
10.4081/cp.2011.e139	313–314	e139
10.4081/cp.2012.e1	1	e1
10.4081/cp.2012.e2	2–4	e2
10.4081/cp.2012.e3	5–6	e3
10.4081/cp.2012.e4	7–8	e4
10.4081/cp.2012.e5	9–11	e5
10.4081/cp.2012.e6	12–13	e6
10.4081/cp.2012.e7	14–15	e7
10.4081/cp.2012.e8	16	e8
10.4081/cp.2012.e9	17–18	e9
10.4081/cp.2012.e10	19–20	e10
10.4081/cp.2012.e11	21–22	e11
10.4081/cp.2012.e12	23–25	e12
10.4081/cp.2012.e13	26–27	e13
10.4081/cp.2012.e14	28–29	e14
10.4081/cp.2012.e15	30–33	e15
10.4081/cp.2012.e16	34–36	e16
10.4081/cp.2012.e17	37–40	e17
10.4081/cp.2012.e18	41–42	e18
10.4081/cp.2012.e19	43–44	e19
10.4081/cp.2012.e20	45–46	e20
10.4081/cp.2012.e21	47–48	e21
10.4081/cp.2012.e22	49–51	e22
10.4081/cp.2012.e23	52–54	e23
10.4081/cp.2012.e24	55–57	e24
10.4081/cp.2012.e25	58–59	e25
10.4081/cp.2012.e26	60–62	e26
10.4081/cp.2012.e27	63–65	e27
10.4081/cp.2012.e28	66–68	e28
10.4081/cp.2012.e29	69–70	e29
10.4081/cp.2012.e30	71–73	e30
10.4081/cp.2012.e31	74–75	e31
10.4081/cp.2012.e32	76–77	e32
10.4081/cp.2012.e33	78–79	e33
10.4081/cp.2012.e34	80–82	e34
10.4081/cp.2012.e35	83–84	e35
10.4081/cp.2012.e36	85–87	e36
10.4081/cp.2012.e37	88–90	e37
10.4081/cp.2012.e38	91–92	e38
10.4081/cp.2012.e39	94–95	e39
10.4081/cp.2012.e40	96–97	e40
10.4081/cp.2012.e41	98–99	e41
10.4081/cp.2012.e42	100–103	e42
10.4081/cp.2012.e43	104	e43
10.4081/cp.2012.e44	105–106	e44
10.4081/cp.2012.e45	108–109	e45
10.4081/cp.2012.e46	108–109	e46
10.4081/cp.2012.e47	110–112	e47
10.4081/cp.2012.e48	113–115	e48
10.4081/cp.2012.e49	116–117	e49
10.4081/cp.2012.e50	118–119	e50
10.4081/cp.2012.e51	120–122	e51
10.4081/cp.2012.e52	123–124	e52
10.4081/cp.2012.e53	125–127	e53
10.4081/cp.2012.e54	128–129	e54
10.4081/cp.2012.e55	130–131	e55
10.4081/cp.2012.e56	132–137	e56
10.4081/cp.2012.e57	138–140	e57
10.4081/cp.2012.e58	141–143	e58
10.4081/cp.2012.e59	144–146	e59
10.4081/cp.2012.e60	147–150	e60
10.4081/cp.2012.e61	151–152	e61
10.4081/cp.2012.e62	153–154	e62
10.4081/cp.2012.e63	155–156	e63
10.4081/cp.2012.e64	157–169	e64
10.4081/cp.2012.e65	161–163	e65
10.4081/cp.2012.e66	164–167	e66
10.4081/cp.2012.e67	168–169	e67
10.4081/cp.2012.e68	170–172	e68
10.4081/cp.2012.e69	173–174	e69
10.4081/cp.2012.e70	175–177	e70
10.4081/cp.2012.e71	178–179	e71
10.4081/cp.2012.e72	180–181	e72
10.4081/cp.2012.e73	182–183	e73
10.4081/cp.2012.e74	184–186	e74
10.4081/cp.2012.e75	187–188	e75
10.4081/cp.2012.e76	189–191	e76
10.4081/cp.2012.e77	192–193	e77
10.4081/cp.2012.e78	194–195	e78
10.4081/cp.2012.e79	196–198	e79
10.4081/cp.2012.e80	199–200	e80
10.4081/cp.2012.e81	201–202	e81
10.4081/cp.2012.e82	203–205	e82
10.4081/cp.2012.e83	206–211	e83
10.4081/cp.2012.e84	212–215	e84
10.4081/cp.2012.e85	216–220	e85
10.4081/cp.2012.e86	221–224	e86
10.4081/cp.2012.e87	225–227	e87
10.4081/cp.2012.e88	228–230	e88
10.4081/cp.2012.e89	231–234	e89
10.4081/cp.2013.e1	1–2	e1
10.4081/cp.2013.e2	3–5	e2
10.4081/cp.2013.e3	6–8	e3
10.4081/cp.2013.e4	9–10	e4
10.4081/cp.2013.e5	11–13	e5
10.4081/cp.2013.e6	14–15	e6
10.4081/cp.2013.e7	16–17	e7
10.4081/cp.2013.e8	18–19	e8
10.4081/cp.2013.e9	20–24	e9
10.4081/cp.2013.e10	25–26	e10
10.4081/cp.2013.e11	27–29	e11
10.4081/cp.2013.e12	30–32	e12
10.4081/cp.2013.e13	33–34	e13
10.4081/cp.2013.e14	35–36	e14
10.4081/cp.2013.e15	37–39	e15
10.4081/cp.2013.e16	40–42	e16
10.4081/cp.2013.e17	43–45	e17
10.4081/cp.2013.e18	46–47	e18
10.4081/cp.2013.e19	48–50	e19
10.4081/cp.2013.e20	51–55	e20
10.4081/cp.2013.e21	56–57	e21
10.4081/cp.2013.e22	58–60	e22
10.4081/cp.2013.e23	61–62	e23
10.4081/cp.2013.e24	63–64	e24
10.4081/cp.2013.e25	65–67	e25
10.4081/cp.2013.e26	68–70	e26
10.4081/cp.2013.e27	71–73	e27
10.4081/cp.2013.e28	74–76	e28
10.4081/cp.2013.e29	77–83	e29
10.4081/cp.2013.e30	84–86	e30
10.4081/cp.2013.e31	87–90	e31
10.4081/cp.2013.e32	91–92	e32
10.4081/cp.2014.599	34–37	599
10.4081/cp.2014.604	1–3	604
10.4081/cp.2014.605	12–14	605
10.4081/cp.2014.608	4–6	608
10.4081/cp.2014.609	10–11	609
10.4081/cp.2014.610	7–9	610
10.4081/cp.2014.628	17–21	628
10.4081/cp.2014.632	15–16	632
10.4081/cp.2014.635	24–26	635
10.4081/cp.2014.637	22–23	637
10.4081/cp.2014.642	50–52	642
10.4081/cp.2014.644	46	644
10.4081/cp.2014.646	43–45	646
10.4081/cp.2014.647	55–57	647
10.4081/cp.2014.648	58–59	648
10.4081/cp.2014.651	30–33	651
10.4081/cp.2014.653	27–29	653
10.4081/cp.2014.656	38–39	656
10.4081/cp.2014.659	47–49	659
10.4081/cp.2014.660	64–65	660
10.4081/cp.2014.661	53–54	661
10.4081/cp.2014.664	40–42	664
10.4081/cp.2014.670	66–69	670
10.4081/cp.2014.679	74–76	679
10.4081/cp.2014.686	60–63	686
10.4081/cp.2014.725	70	725
10.4081/cp.2014.726	71	726
10.4081/cp.2014.738	72	738
10.4081/cp.2014.739	73	739
10.4081/cp.2015.668	3–5	668
10.4081/cp.2015.688	1–2	688
10.4081/cp.2015.697	6–7	697
10.4081/cp.2015.699	11–13	699
10.4081/cp.2015.701	17–19	701
10.4081/cp.2015.706	8–10	706
10.4081/cp.2015.707	37–39	707
10.4081/cp.2015.708	26–27	708
10.4081/cp.2015.717	28–31	717
10.4081/cp.2015.722	14–16	722
10.4081/cp.2015.727	32–34	727
10.4081/cp.2015.733	23–25	733
10.4081/cp.2015.734	40–43	734
10.4081/cp.2015.736	53–55	736
10.4081/cp.2015.740	60–62	740
10.4081/cp.2015.741	50–52	741
10.4081/cp.2015.746	56–57	746
10.4081/cp.2015.748	20–22	748
10.4081/cp.2015.749	47–49	749
10.4081/cp.2015.752	44–46	752
10.4081/cp.2015.754	35–36	754
10.4081/cp.2015.756	58–59	756
10.4081/cp.2015.763	84–87	763
10.4081/cp.2015.767	72–74	767
10.4081/cp.2015.768	78–80	768
10.4081/cp.2015.775	75–77	775
10.4081/cp.2015.776	104–106	776
10.4081/cp.2015.777	94–98	777
10.4081/cp.2015.780	68–71	780
10.4081/cp.2015.781	65–67	781
10.4081/cp.2015.784	88–90	784
10.4081/cp.2015.788	63–64	788
10.4081/cp.2015.792	91–93	792
10.4081/cp.2015.793	124–127	793
10.4081/cp.2015.804	116–118	804
10.4081/cp.2015.810	113–115	810
10.4081/cp.2015.814	99–103	814
10.4081/cp.2015.818	122–123	818
10.4081/cp.2015.819	119–120	819
10.4081/cp.2015.821	107–109	821
10.4081/cp.2016.786	53–54	786
10.4081/cp.2016.811	1–2	811
10.4081/cp.2016.813	13–15	813
10.4081/cp.2016.816	21–23	816
10.4081/cp.2016.820	3–5	820
10.4081/cp.2016.822	6–7	822
10.4081/cp.2016.823	11–12	823
10.4081/cp.2016.824	42–45	824
10.4081/cp.2016.828	16–17	828
10.4081/cp.2016.832	40–41	832
10.4081/cp.2016.837	46–48	837
10.4081/cp.2016.838	18–20	838
10.4081/cp.2016.840	8–10	840
10.4081/cp.2016.841	71–72	841
10.4081/cp.2016.843	27–30	843
10.4081/cp.2016.844	49–50	844
10.4081/cp.2016.846	51–52	846
10.4081/cp.2016.847	24–26	847
10.4081/cp.2016.848	68–70	848
10.4081/cp.2016.849	96–97	849
10.4081/cp.2016.852	55–56	852
10.4081/cp.2016.853	113–116	853
10.4081/cp.2016.855	35–41	855
10.4081/cp.2016.856	38–39	856
10.4081/cp.2016.862	76–77	862
10.4081/cp.2016.865	98–101	865
10.4081/cp.2016.866	81–85	866
10.4081/cp.2016.868	65–67	868
10.4081/cp.2016.871	61–64	871
10.4081/cp.2016.873	57–60	873
10.4081/cp.2016.878	78–80	878
10.4081/cp.2016.879	108–112	879
10.4081/cp.2016.881	73–75	881
10.4081/cp.2016.884	93–95	884
10.4081/cp.2016.885	117–118	885
10.4081/cp.2016.887	89–92	887
10.4081/cp.2016.893	105–107	893
10.4081/cp.2016.895	86–88	895
10.4081/cp.2016.897	122–125	897
10.4081/cp.2016.903	119–121	903
10.4081/cp.2017.857	48–50	857
10.4081/cp.2017.888	1–6	888
10.4081/cp.2017.890	7–10	890
10.4081/cp.2017.898	23–27	898
10.4081/cp.2017.904	31–33	904
10.4081/cp.2017.911	15–19	911
10.4081/cp.2017.912	11–14	912
10.4081/cp.2017.918	61–63	918
10.4081/cp.2017.920	39–40	920
10.4081/cp.2017.921	20–22	921
10.4081/cp.2017.922	64–66	922
10.4081/cp.2017.928	28–30	928
10.4081/cp.2017.930	54–55	930
10.4081/cp.2017.932	36–38	932
10.4081/cp.2017.933	41–44	933
10.4081/cp.2017.936	56–59	936
10.4081/cp.2017.938	45–47	938
10.4081/cp.2017.939	99–100	939
10.4081/cp.2017.942	84–87	942
10.4081/cp.2017.943	66–69	943
10.4081/cp.2017.946	70–71	946
10.4081/cp.2017.950	78–80	950
10.4081/cp.2017.951	75–77	951
10.4081/cp.2017.952	51–53	952
10.4081/cp.2017.953	72–74	953
10.4081/cp.2017.955	96–98	955
10.4081/cp.2017.957	101–103	957
10.4081/cp.2017.958	81–83	958
10.4081/cp.2017.960	88–91	960
10.4081/cp.2017.961	158–159	961
10.4081/cp.2017.969	104–106	969
10.4081/cp.2017.973	124–127	973
10.4081/cp.2017.975	107–110	975
10.4081/cp.2017.976	93–95	976
10.4081/cp.2017.977	117–119	977
10.4081/cp.2017.979	111–113	979
10.4081/cp.2017.982	155–157	982
10.4081/cp.2017.985	148–149	985
10.4081/cp.2017.987	131–136	987
10.4081/cp.2017.992	141–144	992
10.4081/cp.2017.999	137–140	999
10.4081/cp.2017.1002	121–123	1002
10.4081/cp.2017.1006	160–162	1006
10.4081/cp.2017.1008	150–154	1008
10.4081/cp.2017.1009	145–147	1009
10.4081/cp.2017.1018	163–165	1018
10.4081/cp.2018.981	1–4	981
10.4081/cp.2018.991	28–30	991
10.4081/cp.2018.1004	12–14	1004
10.4081/cp.2018.1007	5–7	1007
10.4081/cp.2018.1021	25–27	1021
10.4081/cp.2018.1023	8–11	1023
10.4081/cp.2018.1031	15–18	1031
10.4081/cp.2018.1035	80–81	1035
10.4081/cp.2018.1037	45–46	1037
10.4081/cp.2018.1038	49–51	1038
10.4081/cp.2018.1039	47–48	1039
10.4081/cp.2018.1040	38–41	1040
10.4081/cp.2018.1044	19–20	1044
10.4081/cp.2018.1046	36–37	1046
10.4081/cp.2018.1047	31–35	1047
10.4081/cp.2018.1053	42–44	1053
10.4081/cp.2018.1054	59–62	1054
10.4081/cp.2018.1055	21–24	1055
10.4081/cp.2018.1057	67–68	1057
10.4081/cp.2018.1061	63–66	1061
10.4081/cp.2018.1065	99–101	1065
10.4081/cp.2018.1069	52–54	1069
10.4081/cp.2018.1070	85–87	1070
10.4081/cp.2018.1071	91–93	1071
10.4081/cp.2018.1072	55–58	1072
10.4081/cp.2018.1073	82–84	1073
10.4081/cp.2018.1079	95–98	1079
10.4081/cp.2018.1085	69–72	1085
10.4081/cp.2018.1089	73–79	1089
10.4081/cp.2018.1093	88–90	1093
10.4081/cp.2018.1094	102–103	1094
10.4081/cp.2018.1095	111–113	1095
10.4081/cp.2018.1097	109–110	1097
10.4081/cp.2018.1102	106–108	1102
10.4081/cp.2018.1104	104–105	1104
10.4081/cp.2019.1086	1–3	1086
10.4081/cp.2019.1091	9–11	1091
10.4081/cp.2019.1096	4–5	1096
10.4081/cp.2019.1110	23–25	1110
10.4081/cp.2019.1111	87–89	1111
10.4081/cp.2019.1112	51–54	1112
10.4081/cp.2019.1114	6–8	1114
10.4081/cp.2019.1117	55–57	1117
10.4081/cp.2019.1119	32–37	1119
10.4081/cp.2019.1124	45–47	1124
10.4081/cp.2019.1125	38–41	1125
10.4081/cp.2019.1126	18–22	1126
10.4081/cp.2019.1128	26–28	1128
10.4081/cp.2019.1129	29–31	1129
10.4081/cp.2019.1132	12–17	1132
10.4081/cp.2019.1134	65–68	1134
10.4081/cp.2019.1135	81–83	1135
10.4081/cp.2019.1140	90–95	1140
10.4081/cp.2019.1141	73–74	1141
10.4081/cp.2019.1142	75–77	1142
10.4081/cp.2019.1143	111–113	1143
10.4081/cp.2019.1144	61–64	1144
10.4081/cp.2019.1146	48–50	1146
10.4081/cp.2019.1153	42–44	1153
10.4081/cp.2019.1157	58–60	1157
10.4081/cp.2019.1161	96–101	1161
10.4081/cp.2019.1172	78–80	1172
10.4081/cp.2019.1176	102–103	1176
10.4081/cp.2019.1177	84–86	1177
10.4081/cp.2019.1179	69–72	1179
10.4081/cp.2019.1184	127–132	1184
10.4081/cp.2019.1187	114–117	1187
10.4081/cp.2019.1188	106–108	1188
10.4081/cp.2019.1190	109–110	1190
10.4081/cp.2019.1191	118–122	1191
10.4081/cp.2019.1209	123–126	1209
10.4081/cp.2020.1200	4–7	1200
10.4081/cp.2020.1204	53–55	1204
10.4081/cp.2020.1205	42–46	1205
10.4081/cp.2020.1211	74–77	1211
10.4081/cp.2020.1214	34–36	1214
10.4081/cp.2020.1216	1–3	1216
10.4081/cp.2020.1217	56–58	1217
10.4081/cp.2020.1218	8–11	1218
10.4081/cp.2020.1221	12–14	1221
10.4081/cp.2020.1222	15–16	1222
10.4081/cp.2020.1226	86–88	1226
10.4081/cp.2020.1234	17–19	1234
10.4081/cp.2020.1242	82–85	1242
10.4081/cp.2020.1248	49–52	1248
10.4081/cp.2020.1249	37–39	1249
10.4081/cp.2020.1251	20–23	1251
10.4081/cp.2020.1253	89–92	1253
10.4081/cp.2020.1254	40–41	1254
10.4081/cp.2020.1255	31–33	1255
10.4081/cp.2020.1257	66–69	1257
10.4081/cp.2020.1258	70–73	1258
10.4081/cp.2020.1265	59–61	1265
10.4081/cp.2020.1267	47–48	1267
10.4081/cp.2020.1270	100–102	1270
10.4081/cp.2020.1271	24–30	1271
10.4081/cp.2020.1276	93–95	1276
10.4081/cp.2020.1281	62–65	1281
10.4081/cp.2020.1285	78–81	1285
10.4081/cp.2020.1292	96–99	1292
